# Consequences of Increasing Hypoxic Disturbance on Benthic Communities and Ecosystem Functioning

**DOI:** 10.1371/journal.pone.0044920

**Published:** 2012-10-16

**Authors:** Anna Villnäs, Joanna Norkko, Kaarina Lukkari, Judi Hewitt, Alf Norkko

**Affiliations:** 1 Marine Research Centre, Finnish Environment Institute, Helsinki, Finland; 2 Environmental and Marine Biology, Åbo Akademi University, Åbo, Finland; 3 Tvärminne Zoological Station, University of Helsinki, Hanko, Finland; 4 National Institute of Water and Atmospheric Research, Hillcrest, New Zealand; University of Southampton, United Kingdom

## Abstract

Disturbance-mediated species loss has prompted research considering how ecosystem functions are changed when biota is impaired. However, there is still limited empirical evidence from natural environments evaluating the direct and indirect (i.e. via biota) effects of disturbance on ecosystem functioning. Oxygen deficiency is a widespread threat to coastal and estuarine communities. While the negative impacts of hypoxia on benthic communities are well known, few studies have assessed *in situ* how benthic communities subjected to different degrees of hypoxic stress alter their contribution to ecosystem functioning. We studied changes in sediment ecosystem function (i.e. oxygen and nutrient fluxes across the sediment water-interface) by artificially inducing hypoxia of different durations (0, 3, 7 and 48 days) in a subtidal sandy habitat. Benthic chamber incubations were used for measuring responses in sediment oxygen and nutrient fluxes. Changes in benthic species richness, structure and traits were quantified, while stress-induced behavioral changes were documented by observing bivalve reburial rates. The initial change in faunal behavior was followed by non-linear degradation in benthic parameters (abundance, biomass, bioturbation potential), gradually impairing the structural and functional composition of the benthic community. In terms of ecosystem function, the increasing duration of hypoxia altered sediment oxygen consumption and enhanced sediment effluxes of NH_4_^+^ and dissolved Si. Although effluxes of PO_4_^3−^ were not altered significantly, changes were observed in sediment PO_4_^3−^ sorption capability. The duration of hypoxia (i.e. number of days of stress) explained a minor part of the changes in ecosystem function. Instead, the benthic community and disturbance-driven changes within the benthos explained a larger proportion of the variability in sediment oxygen- and nutrient fluxes. Our results emphasize that the level of stress to the benthic habitat matters, and that the link between biodiversity and ecosystem function is likely to be affected by a range of factors in complex, natural environments.

## Introduction

Disturbance to ecosystems has resulted in global declines in biodiversity [1]–[2]. This has raised concerns since biodiversity is suggested to significantly contribute to valued ecosystem functions, goods and services [3]–[4]. When evaluating the consequences of disturbance for biodiversity-ecosystem function relationships, a central question is what is meant by the term biodiversity. Most biodiversity and ecosystem functioning (BEF) research has focused on species richness alone, excluding other components of biodiversity [5]. However, disturbance to natural communities is known to result in behavioral and compositional changes (e.g. in terms of abundance, biomass and evenness), which precede or accompany species loss [6]. The community degradation pattern depends on species-specific sensitivity towards a particular disturbance, and the resulting non-random change in community composition translates into an altered community performance, which is likely to affect ecosystem functioning depending on what traits are impaired [7]–[9]. Thus, in order to understand how disturbance affects community structure and ecosystem function, it is important to consider more components of biodiversity than species richness alone [8], [10].

In addition to the indirect consequences of disturbance for ecosystem functions, which are mediated by changes in the biota, disturbance may also have direct impacts on the ecosystem. For example, physical disturbances such as fire directly affect net ecosystem production (NEP) through emissions, but NEP is also indirectly affected due to reductions in productivity as functional leaf area is lost [11]. Similarly, sediment deposits due to soil erosion might directly change processes such as benthic primary production and nutrient cycling, but these are also affected by disturbance-induced changes in the benthic fauna [12]. Hence, when striving to evaluate what we lose in terms of ecosystem functions, studies considering both the direct and indirect consequences of relevant disturbance scenarios in natural environments are required [13]–[15].

Marine soft-sediments are among the most common habitats on earth, sustaining highly diverse benthic communities [16]. These communities play an important role in ecosystem functioning, through habitat engineering, by affecting nutrient cycles and primary productivity, and by being an essential part of the food web [17]–[18]. The function of these environments is threatened by a range of anthropogenic stressors, such as over-fishing, habitat destruction, pollution and eutrophication [19]. Such disturbances cause a non-random loss of species [20], which translates into a loss of benthic functionality, depending on what trophic levels [21], functional attributes [22], and interactions [17] are affected. There is, however, still limited empirical evidence on how disturbance-induced changes in benthic communities impact ecosystem function within natural environments (but see e.g. [12], [17], [23]–[24]), which is surprising given that disturbance-mediated species loss is the one of the main reasons for the interest in BEF relationships [12], [24].

Hypoxia (<2 mg O_2_ l^−1^ [25]) is a global key stressor disturbing estuarine and marine benthic ecosystems, affecting >245 000 km^2^ of the seafloor in coastal zones [26] and is predicted to increase due to ongoing human-induced eutrophication and global warming [25]–[28]. The magnitude of hypoxia is highly dynamic, and may vary in extent, severity and frequency. For example, the duration of hypoxia may last hours (dial cycles; [29]), days to weeks (aggregated, drifting algal mats; [30]–[31]) and even months to years (thermal or salinity stratification; [32]). Hypoxia affects the benthic habitat directly by changing diagenetic pathways, the sediment redox-cascade and the direction and magnitude of nutrient fluxes at the sediment-water interface [33]. The benthic response depends on the magnitude and scale of the disturbance, the habitat type as well as species-specific vulnerability and intra- and inter-specific interactions [34]. Initially, oxygen deficiency changes the physiology of the benthos, seen as, e.g., conservation of energy through metabolic depression and growth reduction [35]. Behavioral responses include movement from oxygen-poor areas, stretched out bivalve siphons, abandonment of tubes and burrows, and reduced burrowing depths [36]–[37]. Sub-lethal and lethal oxygen tolerance levels vary greatly between species [27], [38], but a degradation of the benthic community generally follows increased hypoxic stress [39]. When disturbance exceeds the tolerance level of the majority of species, the community might exhibit a threshold response, defined as the point beyond which severe changes in diversity, composition, and function occur, forcing the system into an alternative state [26], [40]. Such a shift is likely to impair community contribution to ecosystem function and might lead to decreased ecosystem resilience and a hysteresis-like recovery of the system [26], [41]. However, experimental *in situ* studies identifying degradation patterns in response to increasing disturbance are scarce [34], hampering the forecasting of shifts in resilience.

An important ecosystem function affected by the benthic community is sediment nutrient cycling. Benthic community functions, such as sediment reworking, bioirrigation and digestion of organic matter, change sediment properties, enhance transportation of particles and solutes, and increase the depth of the redox potential discontinuity (RPD) layer. The contribution of the individual species to such processes depends on what functional characteristics it expresses [14], [42]. For example, undisturbed benthic communities might have deeper-burrowing species [20] that create and ventilate burrows or tubes, which enhance sediment oxygen penetration and stimulate microbial growth and activity. This creates environments where nitrogen mineralization and transformation is enhanced [43]–[44] and sites where phosphate might be adsorbed [45]–[46]. Such positive effects of benthos on nutrient removal and sequestration might be eliminated in stressed communities, which are often dominated by small, surface-dwelling taxa that contribute to a more rapid remineralization of organic matter, releasing nutrients to the overlying water [47], thus impairing the natural purification capability of the sediments [48]–[49].

In BEF research, more studies encompassing the complexity of natural ecosystems and thus the realistic consequences of disturbance-mediated species loss are needed [14], [41]. The multiple factors and feedback loops affecting the relationship between hypoxia, the macrobenthic biota, and sediment ecosystem functioning make it important to evaluate the consequences of disturbance under field conditions (cf. [14], [50]). Nevertheless, few field-studies have been performed in submerged coastal habitats, where hypoxia has become an increasing nuisance [25]. We exposed an *in situ* natural benthic community to increasing duration of hypoxia to evaluate its impact on the benthic community and ecosystem functioning. We performed the experiment in an area where benthic structural and functional diversity is naturally low [51] and the relation between community composition and performance is likely to be pronounced [52]. We predicted that increasing duration of hypoxia would 1) gradually impair different aspects of the benthic community (behavior, species richness and structure), resulting in 2) concomitant changes in benthic biological traits. In addition, we hypothesized that 3) both the direct and indirect effects of hypoxia (i.e. the disturbance-mediated alteration in the benthic community) would be of importance for changes in sediment ecosystem functioning (measured as sediment oxygen and nutrient fluxes). For exploring disturbance-induced changes in the biodiversity-ecosystem function relationship, we emphasize that biodiversity, in addition to species richness, includes parameters describing both the structure (e.g. abundance, biomass) and function (e.g. the number of trait modalities, species and evenness within trait modalities) of the benthic community.

## Materials and Methods

### Study area

The experiment site was located in the northern Baltic Sea, in the middle archipelago zone of the Hanko peninsula (59°50′44″N, 23°14′96″E), Finland. The site was 4 m deep and consisted of bare sandy sediments. Salinity in this non-tidal area is about 5.8 and bottom-water temperature was around 17°C during the experiment. The benthic community was dominated by the bivalve *Macoma balthica*, gastropods belonging to the family Hydrobiidae and the polychaetes *Hediste diversicolor* and *Marenzelleria* spp. All necessary permits were obtained for the described field study from Tvärminne Zoological Station.

### Experimental setup

Hypoxia of increasing duration was artificially induced to the benthic habitat by securing black, low-density polyethylene (LDPE) plastic sheets (1 m^2^) to the seafloor. The disturbance simulated patchy hypoxia induced, for example, by drifting algal mats [30]. The sheets were kept in place by metal rods, which were secured with 30 cm metal pegs to prohibit any water exchange. Although the plastics have the drawback of preventing settling of water-column material and benthic primary production, they have proved to be an efficient way of inducing standardized levels of hypoxia in soft-sediment habitats. We checked that hypoxia was induced by measuring changes in oxygen and H_2_S concentrations beneath three independent plastic sheets after 1.5, 3 and 7 days. The thin layer of water beneath the sheets was sampled through (otherwise sealed) tubes situated in the middle of each sheet.

The experiment included four treatments of 0, 3, 7 and 48 days of hypoxia (4 replicates). The replicates were placed in a block design along a 32 m transect, and any plot was separated by at least three meters from the others. Hypoxia was induced for the 48-day treatment in the beginning of June 2008, while the treatments with 3 and 7 days of hypoxia were started in July. The different durations of hypoxia were ended simultaneously, by carefully rolling the plastic away, avoiding disturbing the plots. Sediment color was noted in each plot, and bivalves (shell length >5 mm) at the sediment surface were counted. All manipulations, chamber incubations and subsequent sampling were done using SCUBA.

### Sediment oxygen and nutrient fluxes

Measurements of sediment oxygen and nutrient fluxes were performed with dark benthic chambers. We chose to exclude any effects of primary production, due to logistical restraints and to control for possible differences in light conditions. After the plastics were rolled away, one chamber frame was pressed 6 cm into the sediment in the centre of each plot, resulting in a final enclosed water volume of ca 6 l (area 504 cm^2^, height 11.9 cm). To avoid sampling initial sediment reactions, flushing of sediment was allowed for 14 h. This period allowed the sediment to reach a quasi-stable state, but not a complete re-oxidation [53]–[54]. Incubation was started by installing dark chamber lids and ended 6.5 h later. Water samples were taken from the chambers at the start and end of the incubation. The water was stirred manually with an internal paddle, before the water samples were withdrawn with syringes (200 ml) from a sampling port in the chamber lid. Replacement water from the surrounding water column was supplied through another port that was placed distant from the sampling port. To correct for water column effects, four 1 l dark LPDE bottles were used for *in situ* incubation of ambient water during the experiment. All sampling equipment was acid washed (10% HCl, rinsed with MilliQ-water) prior to use.

The water samples were processed on the boat. For determination of dissolved oxygen, 50 ml was fixed with 0.5 ml Mn(OH)_2_ and 0.5 ml KI. The rest of each sample was filtered through a Whatman GF/F filter (Ø 25 mm), directly into sample bottles for dissolved Fe (assumed to represent ferroiron (Fe^2+^), 25 ml) and nutrient analyses (125 ml). All samples were stored on ice during transport to the laboratory. Nutrient samples were frozen (−20°C) until further analysis, and 0.83 ml concentrated HNO_3_ was added to the Fe^2+^ samples for preservation before storage at +5°C. Dissolved oxygen concentrations were determined according to the Winkler procedure, while NH_4_^+^, NO_3_^−^ + NO_2_^−^, PO_4_^3−^ and dissolved Si (silicate) were measured spectrophotometrically with an autoanalyser (Lachat QuickChem 8000). The results for NO_3_^−^ + NO_2_^−^ should be interpreted with caution, as concentrations were near the detection limit (NO_2_^−^: 0.06 µmol l^−1^, NO_3_^−^: 0.10 µmol l^−1^). Similarly, the concentration of dissolved iron in the water column was under the detection limit (0.09 µmol l^−1^) as measured with the ICP-OES (Inductively Coupled Plasma-Optical Emission Spectrometry) technique. Concentrations of H_2_S were determined according to Koroleff [55].

### Sediment properties

After incubation, samples for determination of sediment organic matter (OM), total C and N content as well as phosphate sorption properties were taken with cores (Ø 2.0 cm, depth 5 cm). The surface sediment (upper 3 cm) was stored at −20°C for later analyses. Sediment OM was determined as loss of ignition (3 h at 500°C). Sediment samples for nutrient analyses were freeze dried (−70°C) and homogenized. Analyses of TOC and TN were performed with a Carlo Erba high temperature combustion elemental analyzer. Phosphate (PO_4_^3−^) sorption properties of the surface sediments were examined for the 0- and 48-day treatments to clarify the effect of hypoxia on the behavior of phosphate in the sediment-water interface. The PO_4_^3−^ sorption measurements (modification from e.g. Koski-Vähälä and Hartikainen [56]) were carried out by equilibrating 0.50 g of sediment (two replicate samples from each plot) for 24 hours (continuous shaking, 200 rpm, at room temperature) with 25.0 ml of artificial seawater (calculated salinity 5.8) containing 0, 5.0, 10.0, 15.0 and 20.0 µmol l^−1^ of PO_4_^3−^. Supernatants were separated by centrifugation (3000 rpm, 15 min), filtrated (0.4 µm Nuclepore PC membranes) and determined immediately for PO_4_^3−^ by spectrophotometer.

### Macrofauna

After incubation, macrofauna were sampled from each chamber in order to examine their responses to hypoxia and to allow estimation of the possible reduction in their impact on sediment oxygen- and nutrient fluxes. One replicate core (Ø 5.6 cm, depth 15 cm) was taken from the central area of each chamber. Finally, all chambers were excavated, in order to account for any deeper-burrowing bivalves. Macrofauna from disturbed sediments were sieved (0.5 mm), sorted and identified alive to species level at 10× magnification. Samples from undisturbed plots were preserved in 70% ethanol and stained with rose bengal for later analysis. To obtain the proportions of juveniles and adults of dominant taxa, shell lengths of bivalves and gastropods, and the width of the 10^th^ setiger of *Hediste diversicolor* and *Marenzelleria* spp. were measured. Gastropods with <1 mm shell length were only identified to family level. For each replicate, the total weight of each species (precision 0.1 mg blotted wet weight) was determined.

Benthic biological traits were used to investigate how the duration of hypoxia affected the functional structure of the benthic community. The selected traits were considered important for sediment nutrient dynamics, and included benthic feeding modes and aspects of benthic bioturbation. This resulted in a total of five traits summing up to 21 different modalities (the modalities describe possible expressions of a trait; Text S1, Table S1). To examine how the duration of hypoxia affected benthic trait expression, the number of species and Pielou's evenness index were calculated within trait modalities for each treatment. Traits were also combined to a single measure describing the community bioturbation potential (BP_c_ = B_i_^−0.5^*M_i_*R_i_, where B_i_ = size, M_i_ = mobility, R_i_ = reworking mode and position in sediment; modified from Solan et al. [22], when corrected for abundance).

### Bivalve reburial rates

Behavioral changes were assessed by measuring reburial rates of adult *Macoma balthica* in aquaria. Five *M. balthica* were gathered from each replicate plot of the disturbed treatments (n = 20 per treatment) as plastics were removed. As all bivalves in the 48-day treatment were dead, this treatment was not included. The undisturbed treatment was represented by 20 bivalves extracted from unaffected sediments at the study site. In the laboratory, bivalves from each treatment were put in separate compartments of an aquarium (241×23×30 cm), and their reburial rates (minutes) were measured. The aquarium contained 5 cm sediment and water collected from the experiment site, with temperatures similar to field conditions. Oxygen concentrations were saturated throughout the reburial experiment (≥100%).

### Data analysis

One-way analysis of variance (ANOVA) was used to identify differences between treatments for sediment properties, oxygen and nutrient fluxes, if the data fulfilled the requirements of normality (Shapiro-Wilk's test) and homogenous variances (Levine's test). Since no differences in abiotic or biotic parameters could be detected between blocks (p>0.05), block was not included as a factor in the presented analyses. If necessary, data were square root transformed. Any significant differences were further explored with Scheffe's post hoc test and a nonlinear (sigmoidal, logistic, 3 parameter) regression. Size-frequency distributions and reburial rates of *M. balthica* collected from the treatments were compared using Kolmogorov-Smirnov 2-sample tests. Sorption isotherms for sediment phosphate were fitted with a power (3 parametric) regression to the plotted data (x: axis: equilibrium PO_4_^3−^ concentration, y-axis: desorbed/adsorbed phosphate). Differences between curves were indentified with an F-test.

Multivariate analyses of benthic community data were performed with the PRIMER software [57]–[58]. Bray-Curtis measures were based on square root transformed abundance and biomass data in order to down-weigh dominance. Dummy species were included in the analyses to enable calculation of the measure over blank or nearly empty samples. Dummy values force blank samples to a similarity of 100%, which is appropriate in cases when such observations are the consequence of the same cause, e.g. a specific disturbance [59]. One-way analysis of similarities (ANOSIM) was used to identify differences in benthic abundance and biomass between treatments. The SIMPER procedure identified species contributing to (dis)similarities between and within treatments. Comparisons of trait composition between treatments were based on an abundance-corrected traitmatrix (Text S1) and analyzed with one-way ANOSIM [57].

Finally, the relationship between the benthic community and ecosystem functioning over the stress gradient was tested, with the aim of separating out the responses in ecosystem functioning driven solely by the duration of hypoxia, the benthic community, and the indirect effect of hypoxia mediated by effects on the benthic community. To accomplish this, we used multivariate analyses. Ecosystem functioning was defined as all chemical fluxes, and a similarity matrix based on Euclidean distances of normalized variables constructed. The benthic community was defined as number of species, evenness, number of modalities, average number of species per modality, total abundance and total biomass and a Principal Component Analysis (PCA) was then run on the similarity matrix of Euclidean distances of the normalized data. This analysis had the effect of removing the colinearity that existed between some variables and producing a reduced number of variables to represent the benthic community, i.e., the first three axes of the Community PCA (CPCA) which together explained >90% of the variability in the PCA. Following this, a regression-based multivariate analysis (DISTLM, [60]), which includes a randomization procedure, was run to relate the ecosystem function similarity matrix (EFM) to the CPCA scores and the duration of hypoxia, using selection of important variables based on Akaike's Information Criteria. Initially, variables were included as linear factors, and then again including non-linearity as log-transformations and polynomial terms. However, non-linear terms did not improve the amount explained by the models (overall r^2^ terms), so they were not used. Three models were run: (i) EFM predicted by CPCA alone; (ii) EFM predicted by hypoxia alone; and (iii) EFM predicted by CPCA, after the effect of hypoxia had been removed. The results from the three models were then used to calculate the amount of variability explained by the benthic community and hypoxia alone, and the intersection of effects related to the benthic community and the duration of hypoxia, according to Borcard et al. [61]. In order to assess the potential for day 48 to overwhelm our results, an additional DISTLM analysis was run (as above) where the most severely impacted treatment (i.e. the 48-day treatment with no fauna) was excluded. At last, the PCA scores that were significantly related to ecosystem functions were correlated with the included benthic parameters, to distinguish which benthic parameters were most important for explaining ecosystem functions.

## Results

### Hypoxia-induced habitat change

The sandy sediment was dominated by grain sizes between 0.063–0.5 mm [62]. The oxygen saturation rapidly reached hypoxic levels (≤2 mg O_2_ l^−1^) beneath the plastic sheets (the O_2_ saturation was 0.5±0.1 mg l^−1^ after 1.5 days and ≤0.1 mg 1^−1^ after 3 days). After seven days, anoxic conditions (i.e. 0 mg O_2_ l^−1^) and formation of H_2_S (approx. 3 µmol l^−1^) were observed. Visual observations of sediment color confirmed the decrease in sediment oxygenation with increasing duration of hypoxia. The sediment in the 0- and 3-day treatments was light-colored, while sediments exposed to 7 days of deoxygenation had a partially black sediment surface, probably due to precipitation of ferrosulphides under reducing conditions. An intensely black sediment surface, indicative of complete anoxia, was observed in the 48-day treatment where the sediment also was slightly more compacted than the undisturbed sediments. Sediment content of OM, TC and TN were low, and averaged 0.58±0.05, 0.19±0.02 and 0.02±0.00%, respectively. Sediment OM increased in the 48-day treatment compared to the 0- and 3-day treatments, with 13 and 5%, respectively (p = 0.002, Table S2). The 3-day treatment also had slightly higher OM content compared to the 7-day treatment (p = 0.002; Table S2).

### Observations of changes in faunal behavior

**Figure 1:**
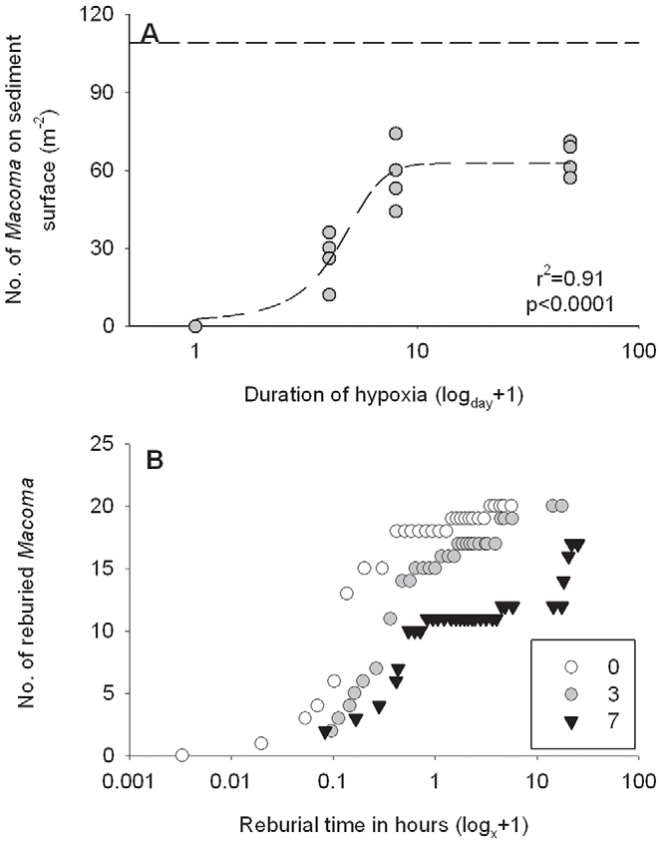
The effects of hypoxic stress on the behavior of *Macoma balthica*. (A) Number of stressed and/or dead *M. balthica* on the sediment surface with increasing duration of hypoxia. A nonlinear regression curve was fitted to the replicate values (r^2 = ^0.92, p<0.0001, Table S2). The dotted horizontal line represents the number of *M. balthica* found at depth in undisturbed sediments. (B) The reburial rate of *M. balthica* after 0, 3 and 7 days of hypoxia. 20 bivalves were included for each treatment (tested in the laboratory). The x-axes are log (x + 1) transformed.

No benthic animals were noted on the surface of undisturbed sediments. Behavioral stress responses were observed in fauna exposed to 3 days of hypoxia, as several siphons of adult *Mya arenaria* protruded out of the sediments, and numerous *Macoma balthica* and polychaetes (*Hediste diversicolor* and *Marenzelleria* spp.) had emerged to the sediment surface. Some dead individuals were noted. After 7 days of hypoxia, a larger number of bivalves and polychaetes were observed, and over half of them were dead or flaccid. In the 48-day treatment, only dead bivalves and empty shells remained. Changes in behavior due to increasing duration of hypoxia are illustrated quantitatively by the increasing number of *M. balthica* observed on the sediment surface (Fig. 1A, Table S2). These numbers can be compared with undisturbed sediments, where no *M. balthica* were observed at the surface, while 109±17 *M. balthica* (>5 mm) m^−2^ were found at depth (the figure is based on core sampling and excavation of flux chambers). The increased stress also resulted in decreasing bivalve reburial rates (Fig. 1B). The majority of undisturbed *M. balthica* reburied within 5 minutes, while bivalves exposed to 3 days of hypoxia reburied significantly slower (K-S test: D = 0.500, p = 0.025). The bivalves exposed to 7 days of hypoxia had the slowest reburial rate, which differed significantly from the other treatments (0–7: D = 0.875, p<0.001, 3–7: D = 0.625, p = 0.002; Fig. 1B).

### Changes in benthic community composition

**Figure 2:**
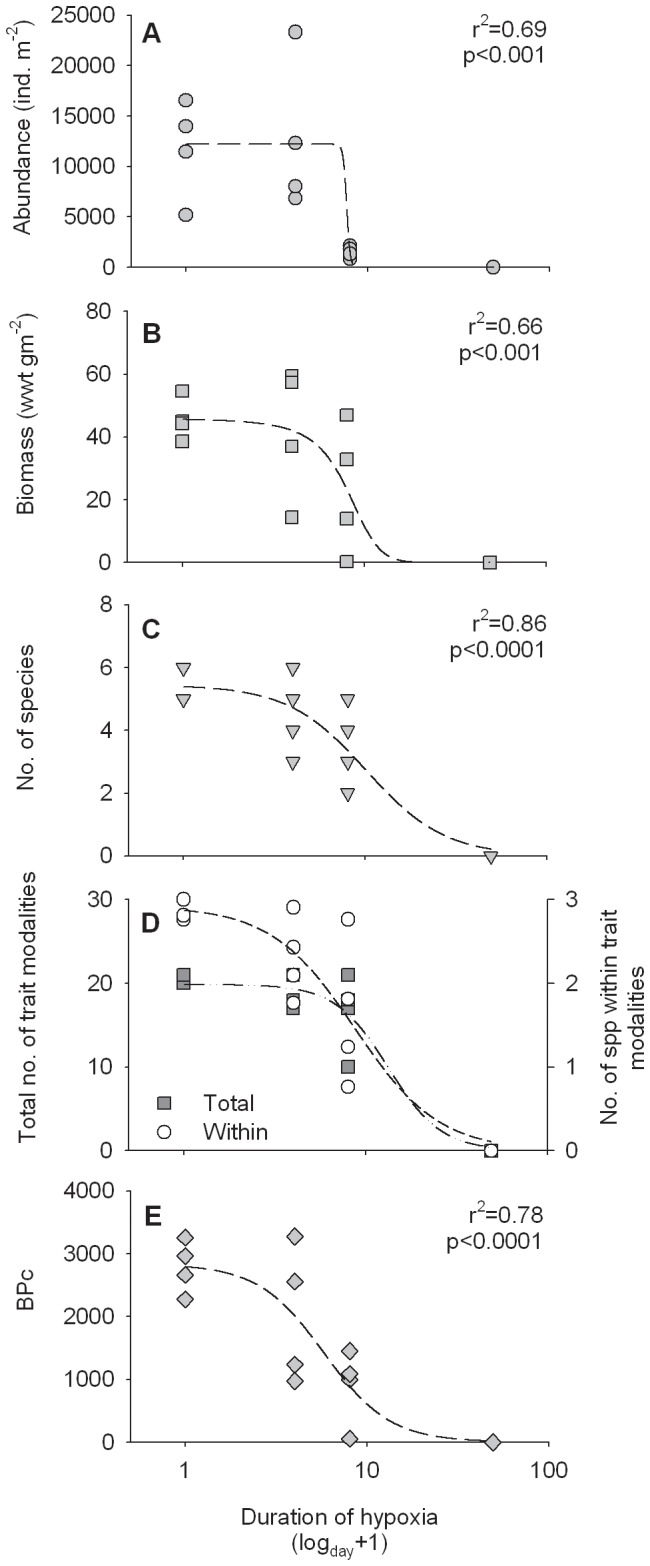
The effect of increasing duration of hypoxia on benthic parameters. (A) abundance (B) biomass (C) number of species (D) total number of trait modalities present (filled squares, primary y-axis, r^2 = ^0.93, p<0.001) and the average number of species within trait modalities (white circles, secondary y-axis, r^2 = ^0.86, p<0.001) and (E) the community bioturbation potential (BP_c_). *Mya arenaria* is excluded from the biomass data. Non-linear regression curves were fitted to the plotted data (Table S2). For presentation, the x-axes are log (x + 1) transformed.

Many aspects of the benthic community exhibited a non-linear response to the increasing duration of hypoxia (Fig. 2, Table 1). The most pronounced decline was observed for benthic abundance, as the relatively high abundances in sediments exposed to ≤3 days of hypoxia were abruptly reduced in the 7-day treatment, and no fauna was observed in sediments exposed to 48 days of deoxygenation (Fig. 2A). For abundance, there were significant differences in community composition between all treatments (Global R = 0.77, p = 0.0001; Table 1). Benthic biomass showed a more gradual decline (Fig. 2B). Large variability was caused by occasional occurrences of the bivalve *Mya arenaria*, which contributed up to 94% of the biomass in a single replicate. When *M. arenaria* was excluded from the data, a significant difference in composition was detected between the 0- and 3-day treatments (Table 1, Fig. 2B). For abundance and biomass, similarity within the 7-day treatment was markedly lower compared to treatments exposed to ≤3 days of oxygen deficiency, while dissimilarities between treatments increased with the duration of hypoxia (Table 1). *Macoma balthica*, *Hydrobia* spp. and *Marenzelleria* spp. contributed most to similarities in both abundance and biomass within the control treatment, while Oligochaeta became important for similarities in abundance after ≥3 days of hypoxia. There were significant differences in the size-frequency distributions of *M. balthica*, separating the juvenile-dominated communities (74% ≤5 mm) in treatments exposed to ≤3 days of hypoxia from the surviving adults in the 7-day treatment (K-S: 0–7, 3–7; D = 0.636, p = 0.017). No difference in size was detected between treatments with ≤3 days of hypoxia (D = 0.182, p = 0.986). The number of species became increasingly variable and slightly declined with increased duration of hypoxia (sigmoidal regression, r^2 = ^0.86, p<0.0001, Fig. 2C, Table S2), but no significant differences were seen between the 0-, 3-, and 7-day treatments (one-way ANOVA; df = 2, F = 3.27, p = 0.085). However, species such as *Hediste diversicolor* and *Cyanophthalma obscura* were only observed in undisturbed sediments.

**Table 1:** Analysis of similarities (ANOSIM) comparing benthic community abundance, biomass and trait composition between treatments (0, 3, 7 and 48 days of hypoxia).

Treatment	Abundance		Biomass*		Traits	
ANOSIM**	R	P	R	p	R	p
Global	0.77	0.00	0.69	0.00	0.81	0.00
0–3	0.57	0.03	0.39	0.03	0.19	0.17
0–7	0.42	0.03	0.22	0.09	0.72	0.03
0–48	1.00	0.03	1.00	0.03	1.00	0.03
3–7	0.44	0.03	0.20	0.06	0.75	0.03
3–48	1.00	0.03	1.00	0.03	1.00	0.03
7–48	0.83	0.03	0.71	0.03	1.00	0.03

SIMPER	Similarity %	Similarity %	Similarity %
0	65.3	79.7	82.9
3	73.4	75.8	79.5
7	25.6	30.9	61.1
48	-	-	-

	Dissimilarity %	Dissimilarity %	Dissimilarity %
0–3	41.0	27.8	21.6
0–7	68.2	50.6	49.7
0–48	100	100	100
3–7	68.7	54.4	51.9
3–48	100	100	100
7–48	100	100	100

	Species contributing to dissimilarites			
0–3	Oli, Mac bal, Man est,	Mac bal, Oli, Hyd spp.,		
	Ost, Mar spp.	Mar spp.		
0–7	Mac bal, Hyd spp.,	Mac bal, Hyd spp., Mar spp.		
3–7	Mac bal, Oli, Hyd spp.,	Mac bal, Hyd spp., Mar spp.		
	Man aes	Oli		

* *Mya arenaria* excluded.

** Dummy sp. included in analyses based on Bray-Curtis measure.

Species abbrevations are; Hyd spp.; *Hydrobia* spp., Mac bal; *Macoma balthica*, Man aes; *Manayunkia aestuarina*, Mar spp.; *Marenzelleria* spp., Oli; Oligochaeta Ost; Ostracoda.

The SIMPER analysis gives the similarities within, and dissimilarities between treatments. Species contributing at least 10% to dissimilarities are listed.

### Changes in benthic trait composition

The total number of benthic trait modalities closely resembled the pattern observed for the number of species (sigmoidal regression, r^2 = ^0.93, p<0.0001, Fig. 2D, Table S2). Interestingly, all modalities were still present in the 7-day treatment (if considering all replicates within this treatment). However, the number of species within single modalities gradually decreased (sigmoidal regression, r^2 = ^86, p<0.0001, Fig. 2D; secondary y-axis, Table S2). Pielou's index of evenness was highest within trait modalities in undisturbed sediments (0.50±0.05) and decreased with increasing stress (3- and 7-days; 0.42±0.07 and 0.32±0.05, respectively).

Benthic trait composition differed significantly between treatments exposed to ≤3 days of hypoxia and those exposed to a longer period of deoxygenation (≥7 days, ANOSIM; Global R = 0.81, p = 0.0001, Table 1). With increasing duration of hypoxia, dissimilarities in trait composition increased compared to undisturbed communities, while trait-similarity within treatments decreased (Table 1). Hence, hypoxia degraded both benthic feeding modes and the community bioturbation potential. Surface detritivores were most abundant in all treatments (>40%), but their contribution became reduced and more variable with ongoing deoxygenation (maximum 66.8±5.8% in undisturbed sediments, minimum 43.2±12.8% in the 7-day treatment). In contrast, the contribution of burrowing detritivores increased from on average 3.0±1.1% in undisturbed sediments, to 20–30% in the 3- and 7-day treatments. The proportional contributions of suspension feeders and herbivores became slightly reduced. The bioturbation potential (BP_c_) of the community showed a non-linear, negative response to increasing duration of hypoxia (Fig. 2E, Table S2).

### Responses in ecosystem function - sediment oxygen and nutrient fluxes

**Figure 3:**
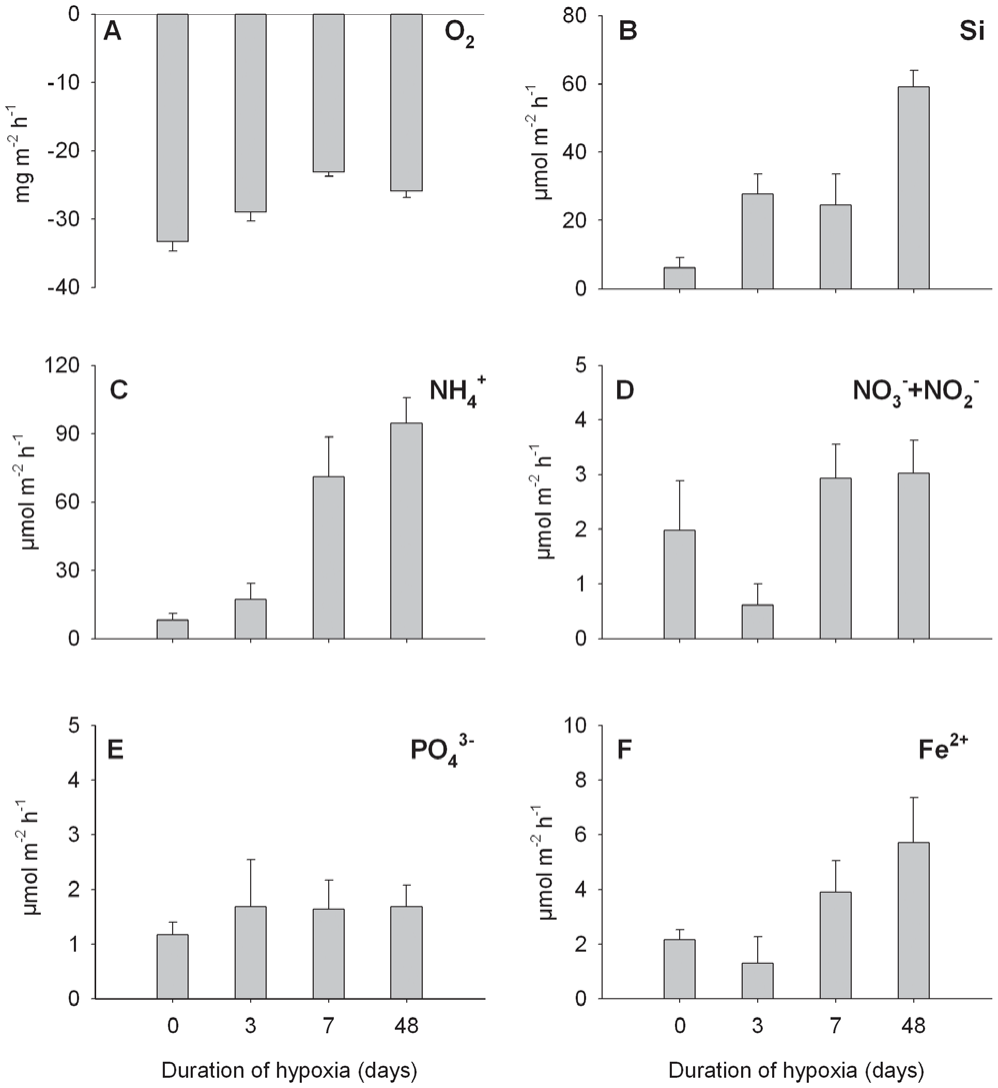
Changes in sediment nutrient fluxes due to increasing duration of hypoxia. The graphs show the average flux (± SE, N = 4) of (A) O_2_ (B) Si (C) NH_4_^+^ (D) NO_3_^−^ + NO_2_^−^, (E) PO_4_^3−^ and (F) Fe^2+^ for each treatment.

During the experiment, the water column had saturated concentrations of dissolved oxygen (9.44±0.03 mg O_2_ l^−1^), with the following concentrations of dissolved nutrients; Si: 7.85±0.09, NH_4_^+^: 0.39±0.24, NO_3_^−^ + NO_2_^−^: 0.05±0.01, PO_4_^3−^: 0.18±0.01, Fe^2+^: 0.07±0.02 µmol l^−1^. The duration of hypoxia significantly affected sediment oxygen consumption and the efflux of silicate and ammonium. Oxygen consumption was reduced with the duration of hypoxia (Fig. 3A), and one-way ANOVA separated the oxygen uptake in undisturbed sediments from that in the 7- and 48-day treatments. Oxygen consumption was also significantly lower in the 7-day treatment compared to the 3-day treatment (p<0.001, Table S2), while a slight increase in oxygen uptake was observed in sediments exposed to 48 days of deoxygenation (Fig. 3A). Oxygen saturation in the chambers was generally over 80%. The flux of dissolved Si was positively related to the duration of hypoxia (r = 0.82, p<0.001; Fig. 3B) and to sediment organic matter (r = 0.59, p<0.05). A significantly stronger efflux of dissolved Si was measured in the 48-day treatment compared to the others (p<0.05, Table S2). The efflux of ammonium increased with the duration of hypoxia (Fig. 3C). For ammonium, sediments exposed to ≤3 days of hypoxia differed significantly from the 7- and 48-day treatments (p<0.001, Table S2). The effluxes of NO_3_^−^ + NO_2_^−^ and PO_4_^3−^ were low, with no significant differences between treatments (Fig. 3D, 3E, p>0.05, Table S2). An increasing trend in the efflux of Fe^2+^ was however noted with increasing duration of hypoxia (r = 0.59, p = 0.015, Fig. 3F), but no significant differences between treatments were observed (p = 0.088, Table S2). The sediment sorption capacity for phosphate was poor, but differed significantly between the 0- and 48-day treatments (F(1, 38) = 6.547, p = 0.015, Fig. S1, Text S2). The PO_4_^3−^ sorption capacity was higher in the 48-day treatment, as the value for equilibrium P concentration (EPC_0_; the x intercept describing the PO_4_^3−^ concentration of the solution where no net desorption or sorption takes place) was lower in this treatment (5.42 µmol l^−1^) than in the control (10.93 µmol l^−1^; Fig. S1, Text S2).

### Relative effects of hypoxia and the benthic community on ecosystem function

**Figure 4:**
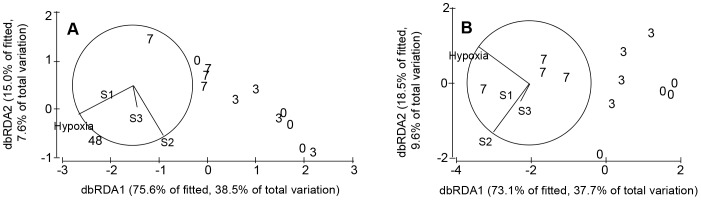
Ordinations illustrating changes in ecosystem functions as directed by the duration of hypoxia and the benthic community. In (A) all treatments are included, while (B) represents the 0, 3 and 7-day treatments. The vector overlays correspond to multiple partial correlations of the predictor variables with the dbRDA axes. Hypoxia depicts the duration of hypoxia (days) while S1–3 depicts the PCA scores used to represent the benthic community.

**Figure 5:**
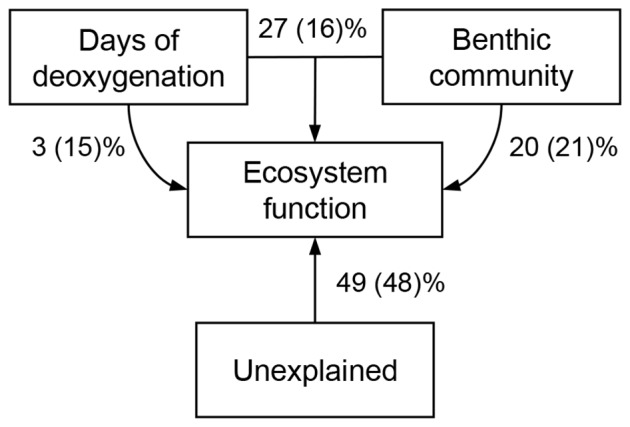
Partitioning of the variation in ecosystem function between the benthic community and the duration of hypoxia. Ecosystem functioning was defined as all chemical fluxes, while the benthic community was described in terms of number of species, evenness, number of trait modalities, average number of species per trait modality, total abundance and total biomass. The variances explained when the 48-day treatment is excluded are within parenthesis.

When including all treatments, the duration of hypoxia and the first 2 axes of the PCA representing the benthic community were individually significantly related to ecosystem function (i.e. sediment oxygen and nutrient fluxes; Table 2, Fig. 4A). Collectively, the included variables explained 51% of the variability in ecosystem function (Fig. 5). Hypoxia alone explained only 3% of the variance, whereas 20% of the variance was explained by the benthic community (i.e. the 3 PCA axes) and 27% was explained by hypoxia and the benthic community together. This latter can be thought of as the indirect effect of hypoxia driven changes in the benthic community. When excluding the 48-day treatment from the analysis, the variables hypoxia and the second PCA axis (S2) were still significantly related to sediment ecosystem function. Partialling out the variance into the different components suggested that the benthic community (i.e. the 3 PCA axes) still could explain 21% of the variance, while 15% was explained by hypoxia alone and 16% by the intersecting effects of hypoxia and the benthic community. In both analyses, the dbRDA ordination indicated that PC axis 2 was the benthic variable that had the largest effect on ecosystem function (Fig. 4A, B, Table 2). The benthic parameters that correlated with PCA score 2 were abundance and biomass (−0.60 < r >0.60, p<0.05) in both ordinations.

**Table 2:** Marginal test results from distance-based linear models (DISTLM) for variables predicting ecosystem function.

	SS(trace)		Pseudo-F		p-value	
Variable	All	Reduced	All	Reduced	All	Reduced
Hypoxia	32.772	23.264	6.352	4.329	0.001	0.001
Score 1	29.794	7.601	5.546	1.095	0.001	0.364
Score 2	16.001	18.159	2.517	3.086	0.040	0.028
Score 3	4.382	2.835	0.610	0.382	0.690	0.882

Ecosystem functions represent fluxes of oxygen and nutrients across the sediment-water interface. Score 1–3 are the first three PCA axes representing the benthic community. Hypoxia is the duration (days) of oxygen deficiency. “All” marks the analysis that includes all treatments, while the 48-day treatment is excluded in the “reduced” analysis.

## Discussion

Healthy communities and a sustained performance of species are essential for retaining the functionality of natural ecosystems. Although disturbance to Earth's ecosystems is increasing, few studies have assessed *in situ* how ecosystem functions are impacted when communities are impaired. By exploring loss scenarios of a natural benthic community in response to increasing duration of hypoxia in field conditions, we show that reductions in community parameters such as abundance and biomass preceded or were concurrent with losses in the number of species. Reductions in benthic trait composition and the benthic bioturbation potential were parallel to the observed degradation in community composition in our natural, low-diverse community. The disturbance-mediated change in community composition was suggested to be an important explanatory variable for changes in sediment oxygen- and nutrient fluxes (foremost in terms ammonium and silicate), but the observed variability in ecosystem function was also directly related to the duration of hypoxia as well as the benthic community.

Numerous studies assessing the consequences of disturbance for ecosystem functioning has focused on the role of declining species diversity. Undoubtedly, the number of species and their identities matter. For example, Waldbusser et al. [63] showed that sediment fluxes and pore-water constituents (O_2_, PO_4_^3−^) differed between multi- and single species assemblages, and that the functional diversity effects were not a simple summation of individual species effects. However, it is indisputable that other factors than the number of species are of importance when evaluating the impacts of disturbance on the biota and ecosystem functioning. For example, the non-random order of species loss is suggested to affect ecosystem function more severely than extinction patterns expected by chance [9], [64]. Abiotic as well as biotic factors and/or interactions have been shown to modify species contribution to ecosystem function [65]–[66] or to affect ecosystem functions directly [13]. The results of our study emphasize that changes in community structure and performance precede or are concomitant with the loss of species. Larsen et al. [8] concluded that in addition to the number of species, changes in abundance and biomass explained the performance of beetles and bees, and were important predictors for changes in ecosystem functions such as dung burial and pollination. Similarly, for organic matter decomposition rates in species-poor stream ecosystems, shredder richness and abundance, especially of particularly efficient species, were found to be the most important explanatory variables [52]. This emphasizes that also other parameters than species diversity should be considered when assessing what we lose in terms of ecosystem functioning when a system is exposed to disturbance.

### Behavioral changes precede community collapse

Behavioral changes are initial macrobenthic responses to hypoxia. The benthic fauna in our study were severely stressed after 3 days of hypoxia, as infaunal polychaetes were observed at the sediment surface, and bivalves had extended their siphons and feet. Emergence at the sediment surface may markedly increase the risk of predation, as demonstrated by Norkko & Bonsdorff [67] and also observed in our study (by perch and flounder). However, macrobenthos may rapidly revert to a normal behavior after short durations of hypoxia [37], and their ability to resume functions promotes benthic ecosystem resilience to and recovery from oxygen deficiency. The rapid reburial rate of *Macoma balthica* exposed to 3 days of hypoxia indicated a sustained performance of this important species. Adult bivalves, which dominated community biomass, are likely to survive shorter periods of hypoxia by closing their valves. A sustained community performance was also supported by the high number of species, abundance, biomass and number of trait modalities still observed in the 3-day treatment. However, the increased variance within these parameters indicated that additional stress might exceed the species' tolerance levels, cf. [68]. Indeed, a more depauperate community was observed in sediments exposed to 7 days of hypoxia (Fig. 2). In this treatment, the reburial capacity of *M. balthica* was reduced, community abundance had declined (a reduction of approx. 88% compared to the control), and benthic biomass, although highly variable, began to decrease. Nevertheless, the number of species was not significantly reduced in the 7-day treatment compared to control sediments. Natural disturbances rarely eliminate all individuals in a community, as perturbations seldom are uniform and as spatial refuges might exist [69]. However, patterns observed in communities exposed to 7 days of hypoxia indicated that the community did lose its adaptive capacity, and species were at risk of becoming functionally extinct *sensu* Dayton [70]. It is also likely that the hypoxic stress was aggravated in treatments exposed to ≥7 of deoxygenation, due to release of hydrogen sulphide [37]. The system experienced a threshold response between 7 and 48 days of hypoxia (as identified by the dbRDA; [71], cf. Fig. 4A). When exceeded, such thresholds are likely to result in altered or lost ecosystem functionality [41].

### Degradation of benthic trait composition

Although low diversity estuarine communities adapted to stress might have a limited number of functional traits, these might be expressed by several species, enhancing community resilience. Identification of the relationship between functional and species diversity is thus critical in order to predict changes in ecological functions following species loss [72]. However, the species within our community did not exhibit much functional redundancy since functional diversity, measured as the total number of trait modalities, closely followed the number of species (cf. Fig. 2C, D). Furthermore, the number of species and evenness within trait modalities was low, and a further reduction was seen with increasing hypoxic duration (Fig. 2D, secondary y-axis). Strong relationships between the number of species and traits or functional groups have also been observed in more diverse assemblages, such as rocky reef communities, fish assemblages, insectivorous birds and Patagonian forbs [72]–[73]. Although such relations are highly dependent on the number of traits included [73], it suggests that species redundancy for some functions might be low even in highly diverse systems [72], emphasizing the importance of the identification and protection of such species and functions.

The relationship between the performance of species and their vulnerability to a perturbation is essential for understanding the effects of disturbance on ecosystem function [12], [74]. While the abundance of traits present describes the potential for benthic performance, the realized performance of individuals can usually not be measured explicitly. Our study showed that oxygen deficiency negatively affected all the considered traits (benthic feeding mode, mobility, size, bioturbation mode and position in sediment; Table S1) long before the community collapsed. Shifts in benthic trait composition were primarily directed by degradation of benthic abundance, which is in line with earlier observations, concluding that shifts in community performance are often more dependent on changes in species densities or identity, than the presence or absence of individual traits [75]–[76]. However, the reduction in trait composition was not entirely homogenous. The proportional contribution of traits expressed by species more resistant to hypoxia, (i.e. adult *Macoma balthica*, *Mya arenaria*, Oligochatea, and *Marenzelleria* spp.; cf. [27]) resulted in a slower decrease of the community bioturbation potential than expected when considering the abrupt reduction in abundance, which was mainly caused by declines of juvenile *M. balthica* and Hydrobiidae. Although such species-identity effects might be crucial for sustaining functioning when facing disturbance [41], the behavioral changes of surviving animals in our study imply that their contribution to ecosystem function was altered. That we could not directly account for changes in behavior when relating benthos to sediment oxygen- and nutrient fluxes probably raised the percentage of unexplained variability in ecosystem function (cf. Fig. 5). Similarly to our results, an overall reduction in trait expression has been observed after dredging [77], with adult sessile non-mobile species the most severely affected. Hence, overall reduction of traits seems common when species have no possibility to avoid a disturbance. Under such scenarios, most biotic parameters are likely to be of importance when assessing the consequences for ecosystem function and focusing on one single parameter or trait is thus not advisable.

### Changes in benthic ecosystem function – consequences of direct and indirect disturbance effects

Disturbances (such as fire, soil erosion) often have evident, direct effects on ecosystem functions, but the indirect changes, mediated by alterations in the biota might also be substantial and complex [11]–[12]. Although field studies have explored benthic responses to different durations of hypoxia (e.g. [30], [47], [78]), few have examined the consequences for ecosystem function. Fluxes across the sediment-water interface give an estimation of alterations in biogeochemical cycling caused directly by a disturbance such as oxygen deficiency (i.e. changes in the sediment redox cascade; [33]), but are also measures of indirect effects, such as disturbance-mediated changes in faunal composition and performance.

In our study, the duration of hypoxia explained only a minor part (3%) of changes in sediment oxygen- and nutrient fluxes (Fig. 5), while a larger proportion of the variability was explained by the benthic community (20%) or by indirect disturbance effects mediated through deoxygenation-driven changes in the benthic fauna (27%). When excluding the 48-day treatment, the proportion explained by the benthic fauna remained the same, while the duration of hypoxia explained a larger part of the overall variability (15%) and the amount explained by hypoxia and benthos together decreased (16%). It is difficult to pinpoint which aspect of the benthic community influenced ecosystem function the most, as all parameters were more or less simultaneously degraded by the hypoxic disturbance. Our results suggest that benthic abundance and biomass, principally directing benthic performance, were of importance.

The benthic fauna is likely to positively affect the release of ammonium from sediments through excretion [79] and through bioturbation, which enhances advection of ammonium produced by bacterial mineralization of organic matter [17]. In our study the flux of ammonium was inversely related to benthic parameters (cf. Fig. 3C and Fig. 2), probably due to ammonification of dead individuals. However, the high bioturbation potential in the 0- and 3-day treatments could also have lowered fluxes of NH_4_^+^ by promoting nitrification, through enlarging the oxic-anoxic transition zone, by transporting solutes into the sediment and by enhancing the rate of microbial processes [44]. It is clear that we cannot separate out the effects of ammonification from changes in bioturbation rates, respiration and excretion.

The low explanatory strength of the duration of hypoxia could partially be due to changes in fluxes during sediment reoxygenation that, in turn, might have contributed to the amount of unexplained variance (49%). For example, the overall degradation of the benthic fauna probably reduced community respiration, which is likely to be the major explanatory factor for the observed decrease in sediment oxygen consumption. However, the increased duration of hypoxia and the co-occurring reduction of the benthic bioturbation potential probably resulted in a reduced RPD-layer [22], as indicated by increasingly black sediments in the 7- and 48-day treatments. Hence, (re-) oxidation processes could be important consumers of oxygen in the 7- and 48-day treatments.

Similarly to ammonium, the efflux of silicate increased with the duration of hypoxia, and showed an inverse relation to benthic parameters (cf. Fig. 3B and Fig. 2). High bioturbation potential may result in a release of silicate [80], which is in accordance with the positive dissolved Si effluxes observed in the ≤3 day treatments. But as silicate also was positively related to sediment organic matter, it is likely that degradation of benthic diatoms had a prominent role for the release of silicate [81], especially in sediments exposed to ≥7 days of hypoxia. Another possible explanation for the increase in dissolved Si after 48-days of hypoxia is that part of the Si is released from surfaces of hydrated oxides of iron [82] as a result of iron reduction. This is supported by the co-occurring increase in dissolved Fe (Fig. 3B and F).

An expected effect in reducing conditions is the reduction of metal ions, such as Fe, coupled to oxidation processes of organic matter or sulfide, and a consequent release of phosphate bound to hydrated oxides of Fe^3+^ [45], [83]. However, we did not observe an increased efflux of PO_4_^3−^ from sediments exposed to hypoxia, probably due to a generally low content of PO_4_^3−^ in this sandy sediment (Text S2, Fig. S1). Nevertheless, as the re-oxidized sediment in the 48-day treatment had a higher capacity to adsorb added PO_4_^3−^ from solution compared to control sediments, this treatment probably induced some PO_4_^3−^ release from ferric compounds (Text S2, Fig. S1, Fig. 3F). As all fluxes were measured after the onset of oxic conditions, re-oxidized iron might have functioned as a barrier at the surface layer, trapping the possible PO_4_^3−^ flux from the sediment [83], a process that has been shown to occur fast, within a day after oxic conditions re-establish [84]–[85].

### Conclusions

We demonstrate that increasing duration of hypoxia gradually impaired the benthic community and caused concurrent changes in ecosystem function (sediment oxygen and nutrient fluxes). Multivariate regression-type analyses suggested that changes in ecosystem function were a result of both direct and indirect disturbance effects, of which the disturbance-mediated changes in the benthic fauna explained a major part. Although our system exhibited some resistance towards the increasing duration of hypoxic stress, the gradual degradation in ecosystem functionality suggested that the system might be more vulnerable than it may appear, and that threshold-like shifts in functionality may take place when species tolerance levels are exceeded (cf. [40], [47]). Our results emphasize that when disturbance scenarios, such as hypoxia, affect all aspects of a community, an integrative research approach, considering the entire community performance as well as the complexity of natural systems (in our case including sediment biogeochemistry) might be fruitful.

In order to provide relevant information considering the effects of disturbance to conservation and management, we have to translate the results from BEF experiments to predictions on how natural communities respond at appropriate scales [15], [17]. In nature, single or several (interacting) stressors are likely to affect both the habitat as well as organisms on multiple trophic levels [21], thus changing existing interactions and feedback loops (e.g. [17]). The importance of investigating the consequences of disturbance in a broader, interdisciplinary context is also emphasized by the fact that one organism may affect multiple ecosystem functions [86], and thus several ecosystem services [87]. Our future understanding of disturbance-induced changes in diversity and subsequent consequences for ecosystem function would benefit from combining results from different spatial and temporal scales, provided by, e.g., observational (monitoring programs), experimental field and laboratory studies [88]. As highlighted by Larsen et al. [8] it may be useful to look at natural communities across disturbed landscapes to examine how losses in biodiversity affects ecosystem function.

## Supporting Information

Figure S1 **Sediment phosphate desorption-sorption behavior after 0 and 48 days of hypoxia at different concentrations of PO_4_^3−^ additions.** Negative values on the y-axis represent desorption from sediment to solution, and the positive values sorption from solution to sediment. Values are given as average ± standard error (N = 4). Isotherms were fitted to replicate values with a power function (r^2^≥0.67, p<0.001, Table S2).
(JNB)

Text S1 **Supporting information considering biological trait analysis.**
(DOC)

Text S2 **Results** **of sediment phosphate desorption-sorption analysis.**
(DOC)

Table S1 **Biological traits depicting benthic feeding modes and qualities important for community bioturbation.** If a species exhibited more than one trait modality, the fuzzy coding procedure was used to assign the species' relative contribution to each modality.
(DOC)

Table S2 **Results** **of nonlinear regression analyses and analyses of variance.** The effects of increasing hypoxic duration on benthic parameters were illustrated with nonlinear regression analyses (sigmoidal logistic 3 parameter equation, c.f. Fig. 1a and Fig. 2). A power function was used to depict the sediment desorption-sorption capacity for PO_4_^3−^. One-way ANOVA was used to detect differences between treatments for abiotic variables.
(DOC)
