# Supplementary material for: Consequences of Increasing Hypoxic Disturbance on Benthic Communities and Ecosystem Functioning
Source: PLoS One. 2012 Oct 16;7(10):e44920. doi: 10.1371/journal.pone.0044920 (PMC3473027; doi:10.1371/journal.pone.0044920)
Supplement: Text S1 — Supporting information considering biological trait analysis. (DOC) [file pone.0044920.s002.doc]

Text S1. Supporting information considering biological trait analysis. The selected biological traits (Table S1) have the potential to affect sediment nutrient cycling. When assigning biological traits to each species, previously published classifications were utilized (e.g. [1]), but also taxonomic and morphologic sources of information (e.g. [2]). A distinction was made between juvenile (< 5 mm) and adult (> 5 mm) *Macoma balthica.* *M. balthica* is fully developed at approx. 2 mm size, when feeding is mediated through the siphon instead of by the foot, while sexual differentiation rarely occurs in individuals < 5 mm [3-4]. Each trait was described by several modalities (or categories; Table S1). To account for the multiple modalities species usually express within a trait, the fuzzy coding procedure [5] was used, allowing species relative affinity to modalities to differentiate, summing up to 1 within a trait [6]. For the trait ‘size’, each species was assigned just to one modality, based on the average biomass of the species at the experiment site. Two trait-data matrices were created. The first species-trait dataset comprised the abundance of each species multiplied by its fuzzy coding for each modality. The second dataset summed up across species, resulting in a single value for each modality in each sample [6].

References:

1. MarLIN BIOTIC; [**http://www.marlin.ac.uk/biotic/**](http://www.marlin.ac.uk/biotic/)

2. Fish JD, Fish S (1996) A Student’s guide to the seashore. Second edition. Cambridge University Press, Cambridge, Great Britain. 564 pp.

3. Caddy JF (1967) Maturation of gametes and spawning in *Macoma balthica* (L.). Can J Zool 45: 955-965.

4. Caddy JF (1969) Development of mantel organs, feeding, and locomotion in postlarval *Macoma balthica* (L.) (Lamellibranchiata). Can J Zool 47: 609-617.

5. Chevenet F, Dolédec S, Chessel D (1994) A fuzzy coding approach for the analysis of long-term ecological data. Freshw Biol 31: 295-309.

6. Hewitt JE, Thrush SF, Dayton PD (2008) Habitat variation, species diversity and ecological functioning in a marine system. J Exp Mar Biol Ecol 366: 116-122.
