# Supplementary material for: Consequences of Increasing Hypoxic Disturbance on Benthic Communities and Ecosystem Functioning
Source: PLoS One. 2012 Oct 16;7(10):e44920. doi: 10.1371/journal.pone.0044920 (PMC3473027; doi:10.1371/journal.pone.0044920)
Supplement: Text S2 — Results of sediment phosphate desorption-sorption analysis. (DOC) [file pone.0044920.s003.doc]

Text S2. Results of sediment phosphate desorption-sorption analysis.

Sediment phosphate desorption-sorption behavior after 0 and 48 days of hypoxia were examined by adding different concentrations of PO_4_^3-^ additions (Fig. S1). The measurements partly explain why the expected release of phosphate bound to hydrated oxides of Fe^3+^ could not be observed in our experiment. This could be due to that the concentration of PO_4_^3-^ in the sediment pore water was low initially, which is supported by the small concentration of PO_4_^3-^ released from sediment to artificial seawater with no added PO_4_^3-^ (Fig. S1). The sandy sediment, poor in OM, probably contained a low amount of poorly crystallised oxides of iron (and aluminium), which are effective in binding phosphate. This is supported by the poor ability of the sediment to bind added PO_4_^3-^ from the solution (Fig. S1). However, some iron-bound PO_4_^3-^ was present in the sediment and prolonged hypoxia reduced ferric iron into soluble ferro-form, which was seen as an increase in Fe^2+^ with the duration of oxygen deficiency (Fig. 3f). Despite the low concentrations, the 48-day hypoxia probably induced PO_4_^3-^ release from ferric compounds. This assumption is supported by the improved ability of the re-oxidized sediment to adsorb added PO_4_^3-^ from the solution in the 48-day treatment compared to the control (Fig. S1): hypoxia-induced iron reduction and consequent PO_4_^3-^ release made more sorption surfaces available for added PO_4_^3-^.
