# Supplementary material for: Consequences of Increasing Hypoxic Disturbance on Benthic Communities and Ecosystem Functioning
Source: PLoS One. 2012 Oct 16;7(10):e44920. doi: 10.1371/journal.pone.0044920 (PMC3473027; doi:10.1371/journal.pone.0044920)
Supplement: Table S1 — Biological traits depicting benthic feeding modes and qualities important for community bioturbation. If a species exhibited more than one trait modality, the fuzzy coding procedure was used to assign the species' relative contribution to each modality. (DOC) [file pone.0044920.s004.doc]

Table S1. Biological traits depicting benthic feeding modes and qualities important for community bioturbation. If a species exhibited more than one trait modality, the fuzzy coding procedure was used to assign the species’ relative contribution to each modality.

| **Trait** | **Trait modalities** |
| --- | --- |
|  |  |
| **Feeding mode** | suspension feeder |
|  | surface detritivore |
|  | burrowing detritivore |
|  | carnivore |
|  | herbivore |
| **Mobility** | crawling (sediment surface) |
|  | stationary |
|  | slow movement |
|  | freely motile |
|  | swim/rafter/drifter |
| **Size** | xs (< 0.001 g) |
|  | s (0.001-0.01 g) |
|  | m (0.01-0.1 g) |
|  | l (0.1-1.0 g) |
| **Bioturbation mode** | tube-dweller |
|  | gallery diffuser |
|  | bio diffuser |
| **Position** | epifauna |
|  | infaunal (top 2 cm) |
|  | infaunal (2-5 cm) |
|  | infauna > 5 cm |
