# Supplementary material for: Consequences of Increasing Hypoxic Disturbance on Benthic Communities and Ecosystem Functioning
Source: PLoS One. 2012 Oct 16;7(10):e44920. doi: 10.1371/journal.pone.0044920 (PMC3473027; doi:10.1371/journal.pone.0044920)
Supplement: Table S2 — Results of nonlinear regression analyses and analyses of variance. The effects of increasing hypoxic duration on benthic parameters were illustrated with nonlinear regression analyses (sigmoidal logistic 3 parameter equation, c.f. Fig. 1a and Fig. 2). A power function was used to depict the sediment desorption-sorption capacity for PO4 3−. One-way ANOVA was used to detect differences between treatments for abiotic variables. (DOC) [file pone.0044920.s005.doc]

Table S2. Results of nonlinear regression analyses and analyses of variance. The effects of increasing hypoxic duration on benthic parameters were illustrated with nonlinear regression analyses (sigmoidal logistic 3 parameter equation, c.f. Fig. 1a and Fig. 2). A power function was used to depict the sediment desorption-sorption capacity for PO_4_^3-^. One-way ANOVA was used to detect differences between treatments for abiotic variables.

| **Nonlinear regression** | **df** | **SS** | **F** | **p** | **r^2^** |
| --- | --- | --- | --- | --- | --- |
| No. of *Macoma* on sediment surface | 3 | 32685.0 | 75.3 | < 0.0001 | 0.92 |
| Abundance | 3 | 1201949375.1 | 14.2 | < 0.001 | 0.69 |
| Biomass (*Mya* excluded) | 3 | 17585.3 | 12.5 | < 0.001 | 0.66 |
| No. of species | 3 | 250.5 | 38.5 | < 0.0001 | 0.86 |
| No. of trait modalities | 3 | 4133.8 | 85.4 | < 0.0001 | 0.93 |
| No of spp. within trait modalities | 3 | 65.0 | 39.4 | < 0.0001 | 0.86 |
| BPc | 3 | 50580848.8 | 22.7 | < 0.0001 | 0.78 |
| PO_4_-P: 0-day treatment | 3 | 0.2 | 17.2 | < 0.0001 | 0.67 |
| PO_4_-P: 48-day treatment | 3 | 0.5 | 42.6 | < 0.0001 | 0.83 |
| **one-way ANOVA** | **df** | **SS** | **F** | **p** | **Scheffe's post hoc** |
| Organic matter | 3 | 0.2 | 8.8 | 0.002 | 0-48*, 3-7*,48** |
| Oxygen flux | 3 | 2.0 | 15.2 | 0.000 | 0-7**,48**, 3-7* |
| Silica flux | 3 | 5828.2 | 13.2 | 0.000 | 0**,3*,7* - 48 |
| Ammonium flux | 3 | 21026.0 | 14.4 | 0.000 | 0, 3-7*,48** |
| NO_x_ flux | 3 | 15.1 | 2.9 | 0.079 | - |
| Phosphate flux | 3 | 0.1 | 0.2 | 0.907 | - |
| Fe^2+^ flux | 3 | 44.6 | 2.8 | 0.088 | - |
| * p < 0.05, **p < 0.01 |  |  |  |  |  |
